# Travel linearity and speed of human foragers and chimpanzees during their daily search for food in tropical rainforests

**DOI:** 10.1038/s41598-019-47247-9

**Published:** 2019-07-30

**Authors:** Haneul Jang, Christophe Boesch, Roger Mundry, Simone D. Ban, Karline R. L. Janmaat

**Affiliations:** 1 Department of Primatology, Max Planck Institute for Evolutionary Anthropology, Leipzig, Germany; 2 Wild Chimpanzee Foundation, Abidjan, Côte d’Ivoire; 3 Institute for Biodiversity and Ecosystem Dynamics, Faculty of Science, University of Amsterdam, Amsterdam, The Netherlands

**Keywords:** Behavioural ecology, Biological anthropology

## Abstract

To understand the evolutionary roots of human spatial cognition, researchers have compared spatial abilities of humans and one of our closest living relatives, the chimpanzee (*Pan troglodytes*). However, how humans and chimpanzees compare in solving spatial tasks during real-world foraging is unclear to date, as measuring such spatial abilities in natural habitats is challenging. Here we compared spatial movement patterns of the Mbendjele BaYaka people and the Taï chimpanzees during their daily search for food in rainforests. We measured linearity and speed during off-trail travels toward out-of-sight locations as proxies for spatial knowledge. We found similarly high levels of linearity in individuals of Mbendjele foragers and Taï chimpanzees. However, human foragers and chimpanzees clearly differed in their reactions to group size and familiarity with the foraging areas. Mbendjele foragers increased travel linearity with increasing familiarity and group size, without obvious changes in speed. This pattern was reversed in Taï chimpanzees. We suggest that these differences between Mbendjele foragers and Taï chimpanzees reflect their different ranging styles, such as life-time range size and trail use. This result highlights the impact of socio-ecological settings on comparing spatial movement patterns. Our study provides a first step toward comparing long-range spatial movement patterns of two closely-related species in their natural environments.

## Introduction

The ecological intelligence hypothesis has proposed that spatial cognitive abilities have evolved in complex foraging contexts to locate scattered and ephemeral food sources within seasonally fluctuating environments^1–5^. A wide range of animal species from insects to primates use spatial memory for foraging (e.g., bees^6^, birds^7^, elephants^8^, mangabeys^9^, baboons^10,11^, chimpanzees^12,13^), and the variation in their abilities to solve spatial tasks provides a unique opportunity for testing this hypothesis. Large-bodied animals with generally large home ranges likely have a greater need to remember food locations within their natural habitats and to navigate efficiently between them to reduce travel costs^14^. Among apes, humans have especially large home ranges resulting from a semi-nomadic lifestyle, and have unique foraging behaviours in terms of central place provisioning^15,16^, which may have exerted a selective pressure on human spatial abilities. To understand the evolutionary roots of human spatial abilities, researchers have conducted comparative studies on spatial performances in humans and our closest living relatives, the great apes^5,17,18^. Some research showed that humans and great apes perform similarly in physical cognition tasks, and thus argued that humans share basic spatial cognitive skills with great apes^19–22^. Others argued that humans have more developed spatial abilities than great apes due to humans’ more sophisticated ability to travel mentally in time and space, enabling humans to pre- or re-experience their movements^23^. Moreover, humans’ abilities to understand the minds of others and to use spoken language are thought to facilitate joint decision making in humans^20,21,24^ on which path would be most efficient. However, it is still unclear how spatial abilities compare between humans and great apes in real-world foraging contexts.

To date, most comparative research has been conducted in laboratory settings where individuals are restricted in their daily use of space. Although studies in captivity allow for well-controlled experiments^25^, many laboratory tasks have little in common with the spatial challenges that foragers encounter in their natural habitats^17,26,27^ and lack physical self-movement over a large-scale space, which has been shown to be crucial for the development of integrated spatial abilities^28^. Therefore, it is also important to study spatial performances in a natural environment where those skills are used on a daily basis^17,29,30^. In this study, we compared spatial performances of humans and chimpanzees (*Pan troglodytes*) by analysing spatial movement patterns during their daily search for food in a natural environment. Our aim was to expand our knowledge about the extent of both species’ spatial performances, their development, as well as their evolutionary drivers. We selected a population of West African chimpanzees that has already been studied for 40 years in the rainforest of the Taï National Park in Côte d’Ivoire (hereafter, ‘Taï chimpanzees’)^31^. We, in addition, selected a subpopulation of the BaYaka foragers, the Mbendjele BaYaka people (hereafter, ‘the Mbendjele’), who go hunting and gathering in the rainforest of the north-western Republic of Congo. Although human foragers are known to consume more nutrient-dense and higher-caloric food resources than chimpanzees, such as large mammals and fish^15,16^, both the Mbendjele people and Taï chimpanzees selectively forage for plant foods, such as nuts and ripe fruits^32–34^. As human foragers are, Taï chimpanzees are also challenged to travel long distances to visit high-valued food locations using long-term spatial memory^12,13,35–39^. This similarity allowed us to conduct a parallel comparative study on their spatial movement patterns towards specific food locations. The complex spatio-temporal distribution of plant foods in African rainforests furthermore created an ideal context to study the spatial movement patterns to these food sources in both human and chimpanzee populations^4,40–43^.

However, comparing spatial movement patterns of human foragers and chimpanzees in a natural environment is not straightforward, because the two species exhibit differences in their foraging behaviours and lifestyles^15,16,44^. Specifically regarding our two study populations, first, the Mbendjele people have a semi-nomadic lifestyle; they move from camp to camp every few months with little overlap in the range areas between camps, which results in a large life-time range size^45^. The Mbendjele people also spend four to eight months per year in villages to cultivate crops in gardens and trade forest foods with villagers^32,45^. In contrast, the home range of adult Taï chimpanzees covers a relatively smaller area (16–31 km^2^) and shows little seasonal variation^46,47^, and chimpanzees spend most of their adult lives in the same home range^31^. Second, the Mbendjele people collect food and take it back to the camp to process and share (‘central place provisioning’^16,48^), whereas Taï chimpanzees consume food as it is encountered (‘feed-as-you-go’ foraging) and make sleeping nests at variable locations within their territories^31^. Third, the Mbendjele people create a trail system and walk on trails with intermittent travels to off-trail areas to visit specific food locations throughout the day (see Laden^49^ for the description of the Efe people’s trail use in the Ituri forest in DRC, which is similar to that of the Mbendjele), whereas Taï chimpanzees rarely use repeated paths when travelling on the ground quadrupedally^44,50^.

**Figure 1:**
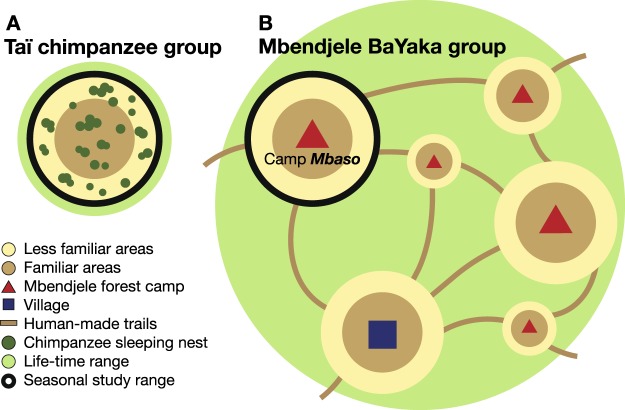
Schematic map of life-time range (*in light green*) and study range (*the black circle*) of (**A**) one Taï chimpanzee community and (**B**) one Mbendjele* band. Within the study range, the central areas tended to be used more regularly and were therefore more familiar (*in light brown*) to the study subjects than peripheral and thus less familiar areas (*in light yellow*). Compared to chimpanzees, the Mbendjele people have a larger life-time range, as every few months they move from one forest camp to another (*red triangles*) using a human-made trail system (*solid brown lines*), but they also stay in a village (*square*) for part of the year. In this study, we investigated spatial behaviours of one Mbendjele band around one forest camp, named Mbaso. *Their ethnic affiliation is referred to as “Mbendjele”^45^, but they often refer to themselves also as “BaYaka”, “Baaka” or “Baka”.

In this study, we first systematically recorded behaviours and ranging tracks of the Mbendjele people and Taï chimpanzees during their daily search for food. Then, to increase the comparability of spatial movement patterns between two populations, we extracted certain travel trajectories from their ranging tracks by following a list of detailed selection criteria. First, we limited our investigation of the Mbendjele’s travel trajectories to the forest area used while the people stayed in one seasonal camp (referred to as ‘the study range’ of the Mbendjele, Fig. 1B), and compared them to the trajectories of Taï chimpanzees in their home range (referred to as ‘the study range’ of Taï chimpanzees, Fig. 1A). Second, we selected travel trajectories of the Mbendjele only when they travelled ‘off-trail’. Third, we selected only terrestrial travel trajectories of Taï chimpanzees. Fourth, we selected travel trajectories toward and away from spatially-stable plants and/or plant-associated foods (e.g., caterpillars, honey, and mushrooms which grow in/on plants) that can be remembered and targeted by foragers from a substantial distance^37^. Lastly, we limited our investigation to female travel trajectories in both populations because we had data of only females for chimpanzees, but also because in this Mbendjele population it is primarily women who gather plant foods and thus move more often between spatially-stable food sources^45^. ‘Off-trail’ travel trajectories represent only a subset of human travels in the forest, but they are most comparable with the data we had for Taï chimpanzees, as human-made trails could largely influence movement patterns (see also Materials and Methods).

To test for evidence of spatial knowledge in movement patterns, we measured the linearity and speed with which the Mbendjele people and Taï chimpanzees travelled along the selected travel trajectories while foraging. A number of studies in both observational and experimental settings have indicated that several primate species including humans increase travel linearity and/or speed when travelling toward known and remembered food locations as compared to unknown or less familiar ones^1,9–11,51–59^. Thus, increased linearity and speed toward out-of-sight food sources are widely used as evidence of anticipation of the food, which implies spatial knowledge and memory about food locations^60^. Foragers of spatially-stable food items can travel in directed paths towards these out-of-sight locations when they possess a mental representation of a number of landmarks (topological map) and/or an accurate coordinate-based cognitive map of the surrounding area (Euclidean map)^61^. In contrast, when foragers possess less accurate spatial knowledge of randomly distributed food locations^59^ or when searching for mobile prey^62^, foragers are likely to engage in undirected searching behaviours, coupled with slow and less linear movements^10,11,59,61^. However, foragers can also reach a food location with high linearity and speed by using only visual cues, without accurate spatial knowledge^9,30^. To avoid these cases, we measured linearity and speed of travel trajectories whose straight-line distance was longer than the visual detection distance of a food source. Our analyses also controlled for possible confounding factors, such as social influence (e.g., group size, female ratio) and motivation (e.g., travelling to ephemeral versus non-ephemeral food locations^38^, food versus non-food location^11^).

We tested two opposing hypotheses: First, humans could travel in off-trail areas faster and more linearly compared to chimpanzees, because a trail system may facilitate humans’ experience with a larger range and this allows humans to develop more accurate spatial mapping abilities. Alternatively, humans could travel in off-trail areas more slowly and less linearly compared to chimpanzees, because humans’ much larger life-time range and their reliance on a trail system may result in less spatial experience with, and thus less accurate spatial knowledge in off-trail areas. To systematically test these predictions, we investigated the potential effects of group composition and familiarity with the respective study range on travel linearity and speed. We predicted that the more familiar individuals are with the area, the better spatial knowledge they have of the food sources in that area, and thus the more linearly and rapidly individuals can move towards food locations. For less familiar areas, however, we predicted that individuals have less spatial knowledge of the environments, and thus are likely to engage more in searching behaviours, which would decrease travel linearity and speed. We further predicted that both Mbendjele people and Taï chimpanzees would increase linearity and speed when they have access to spatial knowledge of a larger number of other individuals in the foraging group, as was found in many other animal species (see Danchin *et al*.^63^, Simons^64^). Both Mbendjele people and Taï chimpanzees live in fission-fusion societies; that is, they forage in groups of variable size in which individuals possess differing spatial knowledge, making it possible to benefit from the presence of others. We expected a stronger effect of the presence of more and/or older group members 1) in less familiar areas, and 2) in humans compared to chimpanzees due to humans’ more sophisticated abilities to make joint decisions using spoken language.

## Results

### Spatial range use of Mbendjele women and female Taï chimpanzees

**Figure 2:**
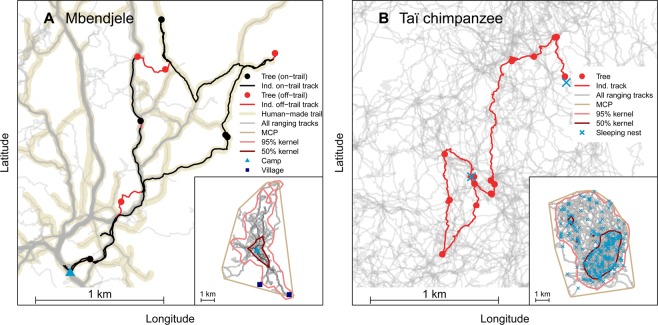
Comparison of range use during daily foraging trips of (**A**) the Mbendjele people and (**B**) Taï chimpanzees. *Zoomed-in maps:* An example of a one-day foraging trip of (**A**) one focal woman *(black and red lines)* and (**B**) one focal chimpanzee *(red line)*, with off-trail trajectories (in *red*) towards and away from off-trail food trees *(red circles)*. *Inlets: Grey lines* show all recorded trajectories during our observation period (Mbendjele: 236 days, Taï chimpanzees: 274 days). The *grey colour intensity* of a trajectory corresponds to its usage frequency *(darker grey* corresponds to a more repeatedly-used path). Range area estimation via minimum convex polygon (MCP, *beige polygons*) and kernel density limits with 95% (*pink*) and 50% (*dark red*) coverage probability. The total lengths of all ranging tracks used to calculate daily travel distance and range size were 1,119 km for five Mbendjele women, and 1,255 km for five female chimpanzees, respectively. The legends are for both the *zoomed-in map* and the *inlet*.

The Mbendjele women had a much larger life-time range of up to 790 km^2^ (estimated through interviews with seven women and forestry maps; Supplementary Fig. S1), compared to Taï chimpanzees who occupied the same home range for most of their lives^46,47^ (~28 km^2^; Table 1). However, the Mbendjele women’s seasonal range around one temporary camp was comparable to the range size of female chimpanzees during a similar number of observation days (Table 1). The Mbendjele women’s average daily travel distance including both on-trail and off-trail travels was also similar to that of Taï chimpanzees (Table 1). In line with reported species differences in foraging styles^15,16,47,48^, the Mbendjele women came back to their seasonal camp after foraging (Fig. 2A), whereas Taï chimpanzees constructed their sleeping nests at different locations within their territories (98% of the nights, Fig. 2B). The Mbendjele women walked 90% of their daily travel distance on human-made trails and only 10% in off-trail areas during their foraging trips (Table 1, Fig. 2A), whereas Taï chimpanzees did not rely on trails for travelling through the forest (Fig. 2B). 74% of the Mbendjele women’s travelling time took place on existing trails (Supplementary Fig. S2B). The Mbendjele’s range use appeared to be more restricted within the commonly used area (95% Kernels), being almost half size of that observed in chimpanzees (Table 1, Fig. 2A). Travelling on human-made trails likely influenced travel linearity (Fig. 2A) and speed (Table 1). When travelling on trails, the Mbendjele women walked more rapidly than chimpanzees, but when travelling off-trail areas the Mbendjele walked with lower speed than chimpanzees (Table 1). The median linearity of the off-trail trajectories towards food locations was very similar in the Mbendjele women and Taï chimpanzees, as was the median travel speed (Table 1).

**Table 1:** Comparison of the overall ranging behaviours of the five Mbendjele women and the five female Taï chimpanzees (above) and the off-trail trajectories analysed in the models of travel linearity and speed (below).

			Mbendjele people (N_days_ = 236)		Taï chimpanzees (N_days_ = 274)
			on-trail travels	off-trail travels	
			Mbendjele people (N_off-trail trajectories_ = 251)		Taï chimpanzees (N_off-trail trajectories_ = 626)
Median daily travel distance (km)			3.93(range: 0.27–13.71)	0.42(range: 0.00–5.23)	4.03(range: 1.11–14.16)
Median walking speed (m/s)			0.76(range: 0.06–2.05)	0.32(range: 0.01–2.84)	0.42(range: 0.07–7.28)
Seasonal range size (km^2^)	MCP 100		30.41		38.65
	Kernel	95%	14.67		27.61
		50%	1.54		6.40
Median linearity^1^			0.87 (range: 0.42–0.99)		0.85 (range: 0.11–0.99)
Median travel speed (m/s)^1^			0.32 (range: 0.06–0.86)		0.44 (range: 0.02–1.10)
Median length of trajectories (m)^1^			101 (range: 10–584)		135 (range: 16–1144)
Median familiarity value of analysed trajectories (minutes)			2.06 (range: 0.34–112.62)		2.28 (range: 0.27–126.91)
Median foraging group size			5 (range: 1–16)		4 (range: 1–21)
Median age of the oldest individual in a foraging group			37 (range: 25–76)		40 (range: 29–52)

^1^Total length of all analysed off-trail trajectories was 35 km for Mbendjele and 116 km for Taï chimpanzees.

### Effects of seasonal familiarity and group composition on travel linearity and speed

**Figure 3:**
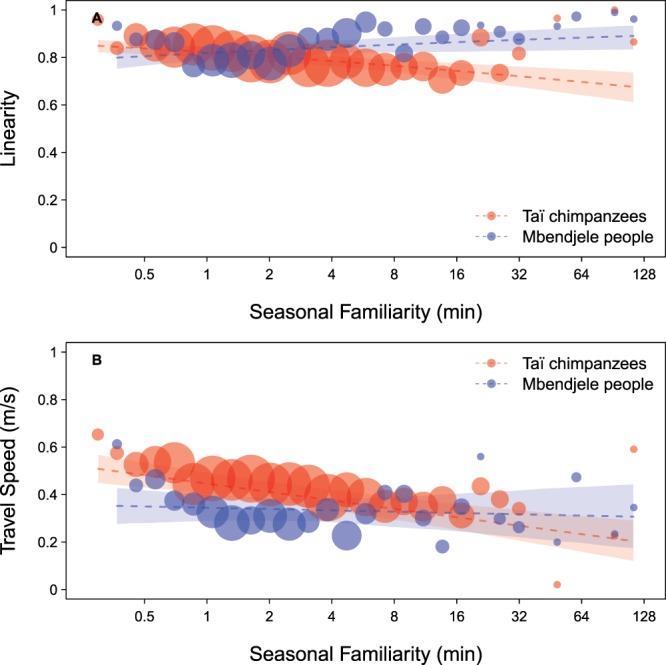
Species differences in the effect of seasonal familiarity with the foraging area on (**A**) travel linearity and (**B**) travel speed. (**A**) The Mbendjele increased linearity as familiarity with the area increased (Estimate = 0.130, Std. Error = 0.066, z value = 1.960, P = 0.050), whereas Taï chimpanzees decreased linearity (Estimate = −0.174, Std. Error = 0.036, z value = −4.816, P < 0.001). (**B**) The Mbendjele’s off-trail travel speed was unaffected by familiarity with the foraging area (Estimate = −0.008, Std. Error = 0.016, t value = −0.501, P = 0.616), whereas Taï chimpanzees decreased travel speed as familiarity increased (Estimate = −0.053, Std. Error = 0.010, t value = −5.454, P < 0.001). The *dashed lines* represent the fitted model (with all other predictors being centered); *dots* represent (A) the averaged linearity and (B) the averaged speed per binned familiarity (29 bins with equal widths in log-transformed familiarity), and their area corresponds to the number of travel bouts in the respective bin (N = 1 to 53 per bin). *Shaded areas* represent 95% confidence intervals of the fitted model.

**Figure 4:**
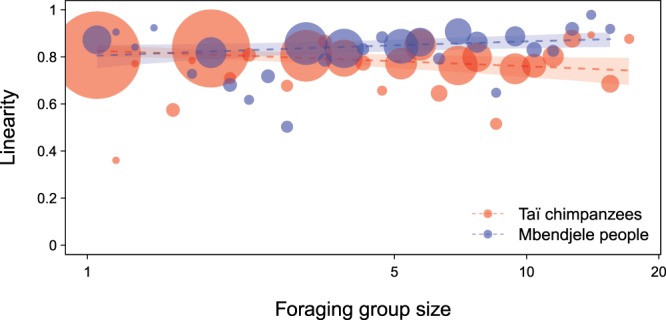
Species difference in the effect of foraging group size on travel linearity. The Mbendjele increased linearity when travelling in a larger foraging group (Estimate = 0.156, Std. Error = 0.071, z value = 2.195, P = 0.028), whereas Taï chimpanzees decreased linearity when travelling in a larger group (Estimate = −0.140, Std. Error = 0.071, z value = −1.969, P = 0.049). The *dashed lines* represent the fitted model (with all other predictors being centered); *dots* represent the averaged linearity per binned group size (29 bins with equal widths in log-transformed group size), and their area corresponds to the relative number of travel bouts in the respective bin (N = 1 to 184 per bin). *Shaded areas* represent 95% confidence intervals of the fitted model.

The comparison between full and null models revealed that there were clear differences between Mbendjele people and Taï chimpanzees (linearity model 1 with group size: *χ*^2^ = 27.590, df = 4, *P* < 0.001; linearity model 2 with maximum age: *χ*^2^ = 20.556, df = 4, *P* < 0.001; travel speed model: *χ*^2^ = 9.597, df = 4, *P = *0.048). After removal of the non-significant interactions (see Supplementary Tables S2, S3, S4 for the results of full and intermediate models), we found that the Mbendjele travelled off-trail with increased linearity as familiarity with the foraging area increased, whereas Taï chimpanzees decreased linearity (interaction between species and familiarity; Table 2A; Fig. 3A). The travel speed model, in turn, revealed that the Mbendjele’s off-trail travel speed was unaffected by familiarity with the foraging area. In contrast, Taï chimpanzees decreased travel speed with increasing familiarity (Table 2B; Fig. 3B). Taï chimpanzees’ off-trail travels were faster than those of the Mbendjele people in less familiar areas but they travelled slightly slower than humans in familiar areas (Fig. 3B). In addition, we found that the Mbendjele increased their linearity when travelling in a larger foraging group, whereas Taï chimpanzees decreased linearity when travelling in a larger group (interaction between species and group size in linearity model 1; Table 2A; Fig. 4). However, there was no effect of the age of the oldest individual in a group on travel linearity (linearity model 2; see Supplementary Table S3 for the results of the linearity model with the maximum age). In the travel speed model, we did not find any obvious effects of maximum age or group size on travel speed (Table 2B). In all models, the straight-line distance exerted a significant influence on linearity and speed (Table 2). The effect of the proportion of females in a foraging group was significant only in the linearity model with group size, whereby linearity decreased with a larger proportion of females (Table 2A). The arrival location type did not have any significant effect on either linearity or speed (Table 2; Supplementary Fig S3).

**Table 2:** Travel linearity and speed models: comparison between the Mbendjele people and Taï chimpanzees.

Effect	Estimate	SE	CL_lower_	CL_upper_	χ^2^	df	P
A. Final linearity model with group size							
(Intercept)	1.332	0.066	1.197	1.467			*
Seasonal familiarity^1^	0.248	0.123	0.012	0.495			*
Species (human)	−0.174	0.036	−0.249	−0.100			*
Group size^2^	−0.140	0.071	−0.250	−0.024			*
**Straight line distance**^**3**^	−**0.174**	**0.045**	−**0.266**	−**0.089**	**11.507**	**1**	**<0.001**
**Female ratio**^**4**^	−**0.134**	**0.053**	−**0.233**	−**0.030**	**6.577**	**1**	**0.011**
Arrival location type (food-inspection location)^5^	0.096	0.075	−0.048	0.237	2.160	2	0.201
Arrival location type (trail)^5^	0.134	0.153	−0.142	0.427			*
**Species (human): Seasonal familiarity**	**0.304**	**0.074**	**0.157**	**0.452**	**17.019**	**1**	**<0.001**
**Species (human): Group size**	**0.296**	**0.094**	**0.123**	**0.477**	**9.897**	**1**	**0.002**
B. Final travel speed model							
(Intercept)	0.394	0.028	0.338	0.450			*
Seasonal familiarity^1^	−0.053	0.010	−0.072	−0.033			*
Species (human)	−0.062	0.032	−0.129	0.004			*
Group size^2^	−0.004	0.009	−0.021	0.013	0.178	1	0.673
**Straight line distance**^**3**^	**0.037**	**0.009**	**0.019**	**0.055**	**9.066**	**1**	**0.003**
Female ratio^4^	−0.006	0.009	−0.024	0.011	0.449	1	0.503
Arrival location type (non-ephemeral food)^6^	0.005	0.022	−0.039	0.047	0.329	2	0.848
Arrival location type (trail)^6^	0.018	0.031	−0.047	0.079			*
Maximum age^7^	0.011	0.010	−0.009	0.032	1.096	1	0.295
**Species (human): Seasonal familiarity**	**0.045**	**0.019**	**0.006**	**0.082**	**4.431**	**1**	**0.035**

Statistically significant results appear in bold. CL: confidence limit.

See Supplementary Table S2, S4 for the results of full and intermediate models and Table S3 for the linearity model 2 with max age.

*Not shown due to having a very limited interpretation.

^1,2,3,7^Log- and then z-transformed; mean ± SD of log-transformed values: ^1^5.01 ± 1.04, ^2^1.07 ± 0.79, ^3^4.50 ± 0.80, ^7^3.67 ± 0.20.

^4^Z-transformed; mean ± SD of the original values: 0.78 ± 0.26.

^5,6^Arrival location type was dummy coded with the reference category: ^5^food-collection location, ^6^ephemeral food site.

## Discussion

We compared the spatial movement patterns of Mbendjele foragers and Taï chimpanzees in a large-scale foraging context – in similar tropical rainforest habitats where visual detection is limited and spatial cognitive skills are thought to be crucial for daily survival. Our direct cross-species comparison revealed that average linearity and speed of the Mbendjele people and Taï chimpanzees were similar when travelling off-trail toward and away from food locations in large-scale areas (Table 1). However, we found a clear difference between the Mbendjele people and Taï chimpanzees in the effects of seasonal familiarity and group size on their travel linearity and speed (Table 2; Figs 3 and 4).

Both the Mbendjele people and Taï chimpanzees travelled with about 85% linearity toward out-of-sight locations in seasonally less familiar areas, which were on average 100 to 135 meters away, respectively (Table 1). The availability of visual cues likely facilitates the foragers’ approach to goal locations with high linearity. However, their highly linear terrestrial travels were achieved in the rainforests where fruit trees or landmarks cannot be seen over long distances due to low visibility with closed canopy and heavily impeding vegetation (average detection range in Djoubé forest: 55.4 m, Taï forest: 28.1 m). Crucially, the selected off-trail trajectories were, in addition, all longer than the visual detection distance of each goal location. Therefore, the high levels of linearity are best explained by the possibility that both human foragers and chimpanzees in this study possess an ability to mentally represent space, possibly with a combination of spatial knowledge of goal locations and the use of visual cues, such as landmarks along the way towards such locations. Furthermore, we did not find any significant effects of arrival location type on travel linearity or speed, respectively (Table 2; Supplementary Fig. S3). This suggests that their linearity and speed were not influenced by differential motivation 1) when travelling to food-collection versus food-inspection locations versus a trail, and 2) to ephemeral versus non-ephemeral food locations versus a trail, nor differential needs of spatial knowledge for reaching a trail compared to reaching a tree. Hence, this result indicates that the observed levels of linearity and speed reflect levels of spatial knowledge of the Mbendjele foragers and Taï chimpanzees.

The similar levels of travel linearity of the Mbendjele people and Taï chimpanzees are surprising due to their differences in home range size, central-place provisioning versus ‘feed-as-you-go’ foraging, and trail use (Table 1, Fig. 2). Despite their differences in foraging styles as well as in life history and brain size^15^, both the Mbendjele people and Taï chimpanzees travelled with high levels of linearity. This result allows us to speculate that the challenging forest environment with low visibility and widely distributed food sources might have driven the development of extensive spatial knowledge and memory of food locations in both populations.

However, the Mbendjele people and Taï chimpanzees clearly differed in the manner in which their travel linearity and speed changed with seasonal familiarity with an area. As we expected, the Mbendjele people travelled with higher linearity toward out-of-sight locations when in more familiar areas compared to when in less familiar areas (Fig. 3A). The Mbendjele’s travel speed was, however, not affected by familiarity with an area (Fig. 3B). In contrast, Taï chimpanzees showed the opposite pattern, moving with higher linearity and speed in less familiar areas compared to in familiar areas (Fig. 3A,B). We are aware that the seasonal familiarity used in this study is only a subset of the life-time familiarity of the Mbendjele people and Taï chimpanzees. Thus, it is not straightforward to explain this differential effect of seasonal familiarity on linearity and speed. One possible explanation lays in the differences in life-time range size and trail use between the Mbendjele people and Taï chimpanzees. The Mbendjele women can use at least 10 seasonal camps in their lifetime (see Supplementary Fig. S1), and thus spend less time in one area surrounding a seasonal camp compared to chimpanzees who live most of their adult lives in the same range. Thus, beyond our seasonal familiarity measure, humans’ time spent in less familiar areas over their lifetime is smaller compared to that of chimpanzees. In addition, the Mbendjele women travelled mostly on human-made trails during their foraging trips (Table 1), which further reduces their time spent in less familiar off-trail areas over lifetime. Hence, the decrease of linearity supports the interpretation that Mbendjele women had less spatial experience in less familiar areas, and that they thus searched more for food locations in these areas. We, therefore, speculate that technological advances in humans, such as making a trail system with the use of tools which facilitates long-distance travels in large space, may have had adverse effects on spatial experience of humans in off-trail areas.

Yet a lack of spatial experience is unlikely to explain the reduced linearity and speed of chimpanzees in familiar areas, given that such an explanation would contradict 1) their high level of linearity and speed in less familiar areas (Fig. 3A,B) and 2) results from previous studies that indicated that the same females were able to remember numerous food tree locations throughout their home range^12,13,35–39^. One possible explanation for this could be that chimpanzees endure a higher risk of intergroup encounters with neighboring chimpanzee groups in peripheral areas, which are the less familiar areas for the chimpanzees^65^. Chimpanzees’ spatial strategies to reduce the risk to encounter neighboring groups could be to engage in knowledge-based and goal-directed travels to known food locations in less familiar areas. On the contrary, in familiar areas where chimpanzees are relatively safe, they might be in a more “relaxed” and “flexible” state, which could result in decreased linearity and speed. For example, the chimpanzees could have rested longer in familiar areas, which could have decreased their travel speed or increased the probability that they changed travel goals after resting. This interpretation is consistent with a previous study suggesting that low linearity and slow travel speed do not always indicate a lack of spatial knowledge and memory (e.g., Chacma baboons move less linearly when returning to the single sleeping site on a cliff of which they have good spatial knowledge^11^) (see also ‘*Post-hoc analyses*’ in Supplementary Information).

Finally, we found that the Mbendjele people and Taï chimpanzees differed in the manner with which group size affected travel linearity. We expected that individuals would increase linearity when they have access to spatial knowledge from a larger number of other individuals in the foraging group^63,64^. As expected, the Mbendjele women increased linearity when they travelled in a larger group (Fig. 4). In contrast, Taï chimpanzees decreased linearity when in a larger group (Fig. 4). One might argue that the effect of group size on individual linearity and speed can be an outcome of within-group competition for food sources, caused by high motivation to arrive at food locations earlier than the other group members^58,60^. However, this seems not the case given the opposite pattern we found, in which the chimpanzees, who do not share food as extensively as humans^15,16^ and thus likely have higher competition than humans, decreased linearity when they move with a larger number of group members (Fig. 4). Crucially, group size did not affect travel speed in either species (Table 2B), suggesting that our results cannot be explained by within-group competition.

Therefore, the most plausible explanation is that when the Mbendjele people are foraging in a larger group compared to a smaller group, they are able to pool more individuals’ knowledge and evaluate this knowledge with the help of spoken language, so as to reach food locations with higher linearity. For the Taï chimpanzees, we speculate that they could be safer from the risk of inter-group encounters or predators when in a larger group compared to a smaller group. Thus, the chimpanzees could have been more relaxed, which could result in less linear travels, as was found in more familiar areas (Fig. 3A). Alternatively, a consensus of the group about travel directions may have been more difficult for chimpanzees to achieve without the use of spoken language, leading to less linear travels in larger groups. The absence of a positive effect of group size on chimpanzees’ linearity, however, does not imply that chimpanzees lack the capacity to use the knowledge of other individuals (see Crockford *et al*.^66^, Leeuwen *et al*.^67^). To determine the extent to which chimpanzees can benefit from each other’s spatial knowledge, further research is required in more challenging conditions (e.g., in the context of female emigration).

Overall, our results do not seem to fully support either of our two opposing hypotheses, neither humans travelled faster and more linearly than chimpanzees nor vice versa. Instead, the Mbendjele people and Taï chimpanzees travelled with the similarly high levels of linearity, but their responses to group size and familiarity with an area were significantly different. We acknowledge that our study included only a small sample of human foragers and chimpanzees from a single population, and only females from each population. We are aware that in humans, men and women could have different ranging behaviours and spatial abilities^68,69^, which might impact the results. Moreover, our human data incorporate only a small fraction of their spatial behaviours since we analysed only off-trail travels around one seasonal camp. Nevertheless, our study provides an insight into how two closely-related species in similar environments can differ in their spatial movement patterns, which possibly results from their different ranging styles. This emphasizes that socio-ecological factors should be considered when comparing spatial movement patterns of primate species. Our study shows how a comparative study can be undertaken in natural habitats by selecting data following a detailed list of criteria which can be applied for both species. We expect that this study will contribute to expanding comparative studies on spatial abilities to a wide range of primate species and populations in natural environments.

## Materials and Methods

### Study subjects and data collection

We conducted focal follows of five adult female chimpanzees from the South community (mean community size: 29 individuals, range: 23 to 40) in Taï National Park, Côte d’Ivoire over 274 days from April 2009 to August 2011. The mean number of observations per focal chimpanzee was 26 days (range: 20 to 128 days) over 3 years. Here we used published data on behaviours and daily ranging tracks of Taï chimpanzees^13,36–39^, but for the current study we reanalysed them. We also conducted focal follows of five Mbendjele women from one band (mean band size: 47 individuals, range: 20 to 79) in the forest near the village Djoubé at the Motaba River in the Republic of Congo over 236 days (March to August 2015 and 2016). The mean number of observations per focal woman was 48 days (range: 41 to 53 days) over 2 years. To reduce possible confounding by food availability, we conducted focal follows in observation periods that covered the same fruiting seasons in subsequent years. The data on the two populations were collected in two different geographic locations, however, both Taï and Djoubé forests are flat lowland tropical rainforests with very similar tree density (measured as the number of trees with a DBH larger than 10 cm/ha: Djoubé; 501, Taï; 507, see Supplementary Methods and Janmaat *et al*.^4^, respectively).

To maximize comparability, we collected behavioural data of the Mbendjele by using the same protocol which was used to collect behavioural data for Taï chimpanzees (as in Janmaat *et al*.^13^). We conducted continuous focal sampling^70^ over consecutive days with a Garmin hand-held Global Positioning System (GPS) device. All focal females were fully grown and competent foragers, with the mean estimated age being 36 years (range: 29–39) for Taï chimpanzees and 37 years (range: 29–46) for the Mbendjele, respectively. The location of the focal subject was automatically recorded with a GPS setting ‘as often as possible’ (mean: every 8 seconds, range: 4–36). We on purpose did not record data using regular interval settings to avoid that commercial GPS software influences the accuracy of locational data^13^. We marked all locations where the focal individuals exhibited foraging-related behaviours such as inspecting, collecting, or feeding behaviour. We continuously recorded the foraging group composition^70^. For the Mbendjele, we obtained informed consent from each focal woman and their families, allowing us to follow their daily foraging trips (see also *‘Full details of data collection*’ in Supplementary Methods).

### Calculation of daily travel distance and range size

We cleaned the GPS track data by removing outlying points due to GPS errors, using a program written by R.M. and K.J. (see Appendix in Janmaat *et al*.^13^). Then we removed locations less than one minute apart from the cleaned track data, and calculated total travel distance per day by summing up the distances between the points recorded every minute using R^71^ (version 3.5.0). To calculate range size, we included all GPS tracks and used two widely used methods for range size estimation: the minimum convex polygon (MCP) and the Kernel density estimation, using the packages ‘adehabitatHR’^72^ (version 0.4.14) and ‘maptools’^73^ (version 0.9–2). The minimum convex polygon is the smallest convex polygon enclosing all the locations of the focal individuals (MCP 100). Kernel density estimation quantifies a distribution of the focal individuals’ utilization of a space over the study period^74^. The 95% and 50% kernel correspond to the area in which the probability to relocate the focal individual is equal to 0.95 and 0.50, respectively.

### Selection of travel trajectories to calculate linearity and speed

To measure linearity and speed, we used the cleaned GPS tracks of both Mbendjele people and Taï chimpanzees when moving ‘off-trail’ towards a food source. The reason for including only off-trail trajectories in our analyses of travel linearity and speed was that when walking on a trail, linearity is obviously dictated by the shape of the trail, and humans are considerably faster when travelling on trails. Hence, on-trail trajectories do not more provide information relevant to the aim of our study. A human-made trail can be visually distinguished by the bare soil, repeatedly walked by humans. We continuously recorded on- and off- trail travels when in the forest. Our sample size of the Mbendjele’s travel trajectories between off-trail food locations was limited because the Mbendjele people repeatedly came back to a trail after collecting off-trail foods, then travelled on-trail, and went off-trail again to the next food location (only 81 travels between off-trail food locations compared to 626 travels of chimpanzees). Therefore, we also included the trajectories from a trail towards an off-trail food (61 trajectories) and from an off-trail food location back to a trail (109 trajectories). Because a trail is a long line, we expected it to be easier to reach a trail with high linearity, even if spatial knowledge of the trail location is limited, compared to when individuals reach a food location. To investigate whether such an effect was present in humans, we controlled for the type of arrival location in the models. We defined the departure location of a trajectory as the GPS track point at which individuals initiated their movement towards the next food location after they had finished feeding, collecting, or inspecting food sources at the previous food location or after individuals had left a trail to move towards an off-trail food source. The arrival location of a trajectory was defined as the first GPS track point where the individuals reached the next food location or a trail. We included locations of patchily distributed foods, such as tuber or mushroom patches for the Mbendjele and leaf patches for Taï chimpanzees, to which individuals were observed to return (see *‘Definition of tuber and mushroom patches*’ in Supplementary Methods and Supplementary Table S6). In total, we had 626 trajectories in which chimpanzees arrived at 597 fleshy fruit trees and 29 nut trees, and 251 trajectories in which the Mbendjele people arrived at 65 trees (24 fleshy fruit trees, 22 nut trees, 10 caterpillar trees, and 9 honey trees), 75 tuber patches, 2 mushroom patches, and 109 locations on trails.

Linearity of a travel trajectory was calculated by dividing the straight-line distance between the departure and arrival locations by the distance travelled, which was estimated as the sum of the distances between consecutive points of cleaned GPS tracks^10,12^. The linearity index ranges from close to zero to one. An index of one indicates a completely linear travel between the departure and arrival locations. The average travel speed along a trajectory was calculated by dividing the distance travelled by travel duration between the departure and arrival locations. Resting times were included when calculating travel duration. To avoid cases in which individuals potentially navigated to a goal using only visual cues, we measured the visual detection distance of the most conspicuous food sources (i.e., trees) at the arrival location of each trajectory. Our field assistants revisited the tree location in the following days from the direction in which focal individuals approached the tree, and walked backward with a measurement tape until the tree was not visible anymore (detection range in Djoubé forest (including garden areas): median = 55.4 m, range: 14.5 to 210.0 m, N = 113 trees; Taï forest: median = 28.1 m, range: 5.0 to 62.1 m, N = 2150 trees). We excluded travel trajectories whose straight-line distance was shorter than the respective visual detection distance. When a Mbendjele woman travelled toward a trail, a ground-level food (i.e., mushrooms), or an underground food location (i.e., tubers), we assumed the detection distance was equal to zero and used the average GPS error during travel as the visual detection distance instead (Djoubé forest: 7.56 m, Taï forest: 4.79 m; see Appendix in Janmaat *et al*.^13^).

### Seasonal familiarity with an area within the study range

**Figure 5:**
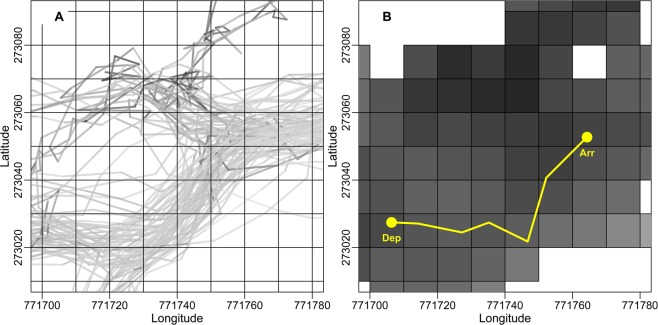
Part of the study range with the 10 × 10 m grid cell system used to determine seasonal familiarity values of travel trajectories. (**A**) Example of grid cells with all GPS tracks (*solid grey lines*) that traversed the grid cells during the observation period. The *grey colour intensity* of a track corresponds to the time spent on the different segments of the track. (**B**) Familiarity value of a trajectory (here in yellow with the departure point ‘Dep’ and the arrival location ‘Arr’) was calculated by averaging the familiarity indices over all grid cells traversed. Darker grey colour in a grid cell corresponds to a longer time spent in that grid cell and nearby grid cells within the detection range of 28.1 m (range: 0–513 minutes).

The Mbendjele have a much larger range that they use over their lifespan than Taï chimpanzees. This made it difficult to obtain data to determine the Mbendjele’s overall utilization and thus overall familiarity in their range. Hence, we used all tracks of five focal individuals to determine a proxy for their familiarity with the study area (hereafter, ‘seasonal familiarity’) and defined it as the time our focal women spent within fractions of the study range during the observation period. To this end, we overlaid a 10 × 10 m grid cell system on the entire study range used by each respective population during our observation period (Fig. 5A). To account for the visibility in the forest, we calculated a ‘seasonal familiarity’ index for each grid cell as the sum of time spent (minutes) in that grid cell plus the time spent in nearby grid cells within the detection range. To make familiarity values comparable, we used the same detection range of 28.1 m from the Taï forest for both the Mbendjele people and Taï chimpanzees. As the detection range is larger in Djoubé forest, this approach is conservative for the humans. As trajectories usually passed through several grid cells with different familiarity indices (Fig. 5B), we calculated for each trajectory the weighted mean^75^ of the familiarity indices of all grid cells traversed. To calculate the weighted mean, we multiplied the familiarity index of each cell that a trajectory traversed and the length of the trajectory in each cell, summed these products, and then divided the sum by the total length of the trajectory. We only used trajectories that passed exclusively off-trail grid cells for analyses. A high level of seasonal familiarity implies a longer time spent in a grid cell during the observation period and thus foragers’ increased experience in that area. The level of seasonal familiarity and distance from the camp were only weakly correlated (Spearman’s rho = −0.277). A trajectory that passed through off-trail grid cells which are close to a human-made trail can be associated with a large familiarity value, which is appropriate as humans are likely more familiar with the areas close to a trail. However, a grid cell being close to a trail is not necessarily associated with a high familiarity value as some trails had been used only rarely during the observation period.

### Group composition

To test for a possible effect of the presence of social partners on the focal individual’s spatial movement patterns, we used group composition as proxy for the accumulated spatial knowledge that the focal individuals can access (hereafter, ‘group knowledge’): 1) the number of independent individuals and 2) the estimated age of the oldest individual in a foraging group. We expected that the presence of a larger number of and/or older group members would increase travel linearity and speed in both human foragers and chimpanzees^64,76^. First, we calculated the weighted mean of the number of individuals present with the focal females during a travel bout of each trajectory (hereafter, ‘group size’), and second, we calculated the weighted mean of the age of the oldest group member present with the focal female during the travel bout (hereafter, ‘the maximum age’). Since the exact birth dates of the Mbendjele people and Taï chimpanzees were unknown, we estimated the age of individuals in the Mbendjele band and Taï chimpanzee community. For chimpanzees, we used the estimated ages of long-term data from the Taï Chimpanzee Project^31^. For the Mbendjele, we conducted detailed interviews with ten Mbendjele people and two Bondongo villagers, and estimated the Mbendjele’s ages by comparing them with the known ages of two villagers of 75 and 44 years old as well as the known ages and inter-birth intervals of Mbendjele children who were born after we began our project in 2014.

### Model explanations

We used linearity and speed as separate response variables because travel linearity and speed were only weakly correlated (Spearman’s rho = 0.267). First, we designed two linearity models with each of the two proxies for group knowledge, namely group size (linearity model 1) and the age of the oldest individual in a foraging group (‘maximum age’; linearity model 2). In the one travel speed model, we used only the maximum age as a proxy for group knowledge, and included group size as a control predictor because within-group competition can also cause an increase in travel speed^58^. All three models included a three-way interaction and all contained two-way interactions between species (humans or chimpanzees), seasonal familiarity, and group knowledge (i.e., group size or maximum age, respectively), since we hypothesized that the effects of seasonal familiarity and group knowledge on linearity and speed would interact with each other and that the pattern of this interaction differs between human foragers and chimpanzees. We controlled for the type of observed behaviour or food type at the arrival location of each trajectory, because different behaviours or food types can result in different foraging or searching strategies, and thus influence travel linearity and speed. For the two linearity models, we categorised the arrival location types into 1) food-collection sites, when foragers collected and/or fed on food items, 2) food-inspection sites, when foragers inspected but did not collect nor feed on food items, and 3) trails, when foragers returned to a trail. By this, we controlled for the cases in which a food-collection location is likely to be reached with higher linearity than a food-inspection location, since the latter likely resulted from searching behaviours^13^. We also controlled for a return to a trail because a trail can be more easily reached with high linearity without spatial knowledge. We had 545 food-collection locations, 223 food-inspection locations, and 109 returns to a trail. For the travel speed model, we categorised the arrival location types into 1) ephemeral food sites, 2) non-ephemeral food sites, and 3) trails, as foragers likely move more quickly toward ephemeral food sources which may be rapidly depleted by competitors (e.g., figs for chimpanzees^38^; caterpillars, ripe fleshy fruits, and mushrooms for the Mbendjele). We had 77 ephemeral food sites, 691 non-ephemeral food sites, and 109 returns to a trail. We opted for including ‘return to a trail’ trajectories and controlling for them in the models rather than excluding them, because the accuracy of the estimates of other fixed and random effects likely improves when the sample size is larger. In all three models, we controlled for the straight-line distance of the trajectories and the proportion of females in a foraging group (see Normand and Boesch^12^). We fitted a *post hoc* model for travel speed which included an additional two-way interaction between group size and species but otherwise identical, to account for the possibility that group size is correlated to competition in chimpanzees more than in humans. However, with regard to our main test predictors, this left conclusions unaltered (see Supplementary Table S7 for the result of this *post hoc* model).

### Statistical analyses

For the two linearity models, we used Generalized Linear Mixed Models (GLMM)^77^ with a beta error structure and logit link function^78–80^. For GLMMs, we used the function ‘glmmTMB’ of the ‘glmmTMB’ package version 0.2.2.0^81^ in R version 3.5.0^82^. For the one travel speed model, we used a linear mixed model (LMM)^77^ with a Gaussian error structure and identity link function. For LMMs, we used the function ‘lmer’ of the ‘lme4’ package version 1.1–19^83^ in R. All three models were based on 877 travel trajectories (251: Mbendjele people, 626: Taï chimpanzees). We included random effects for the identity of the oldest individual in a foraging group, resource species at the arrival location, arrival location identity, and observation day (see Supplementary Table S8 for the results regarding the random effects). We included theoretically identifiable random slopes for the fixed effects within random intercepts, but for glmmTMB models we simplified the random slope structure to solve convergence issues^84,85^ (see Supplementary Table S8). We compared each full model with a respective null model^84^, lacking only species (humans or chimpanzees) and its interaction terms but with an otherwise identical structure, using a likelihood ratio test with the R function ‘anova’^86^. We determined the significance of individual effects by dropping them one at a time^85^ and comparing the models using a likelihood ratio test. We considered P ≤ 0.05 as significant and 0.1 ≥ P > 0.05 as a ‘trend’ to alleviate the issue of dichotomizing decisions about significance at a fixed threshold^87^. We removed non-significant interactions from the models (conditional on the full-null model comparison being significant) and fitted reduced models. All p-values are two-tailed. We determined 95% confidence intervals using the function ‘simulate.glmmTMB’ of the ‘glmmTMB’ package^81^ or ‘bootMer’ of the ‘lme4’^83^ in R. When an interaction between the factor ‘species’ and a covariate (e.g., familiarity) was significant, we conducted *post hoc* tests to infer whether the effect of the covariate differed from zero in either of humans or chimpanzees (see also ‘*Full details of statistical analyses*’ in Supplementary Methods).

### Ethical approval and informed consent

The field research on Mbendjele BaYaka people and Taï chimpanzees was approved by the relevant authorities of the Republic of Congo and Côte d’Ivoire, respectively (Ethics Approval Number for the research on Mbendjele BaYaka people: N°070/MRSIT/IRSEN/DG/DS from the Ministère de la Recherche Scientifique et de l’Innovation Technologique, the Republic of Congo; on Taï chimpanzees: 208/MESRS/DGRSIT/KYS/sac and permission 2008/08_922 from the Ministère de l’Enseignement Supérieur et de la Recherche Scientifique, Côte d’Ivoire). All study procedures were carried out in accordance with the national laws and regulations of the respective country, as well as the ethical standards of the Max Planck Institute for Evolutionary Anthropology and the Primatology department’s ethical guidelines for non-invasive research, and the Comité d’Ethique de la Recherche en Sciences de la Santé (N°095 /MRSIT/IRSA/CERSSA) in Brazzaville, Republic of Congo. For the Mbendjele people, we obtained informed consent from each focal woman and their families, allowing us to follow their daily foraging trips.

## Supplementary information

Supplementary Information

## Data Availability

The data used for this study are available from the corresponding author on request.
