# Supplementary material for: Travel linearity and speed of human foragers and chimpanzees during their daily search for food in tropical rainforests
Source: Sci Rep. 2019 Jul 30;9:11066. doi: 10.1038/s41598-019-47247-9 (PMC6667462; doi:10.1038/s41598-019-47247-9)
Supplement: Supplementary file 1 — Supplementary Information [file 41598_2019_47247_MOESM1_ESM.pdf]

Supplementary Information

***Title*: Travel linearity and speed of human foragers and chimpanzees during their daily search for food in tropical rainforests.**

Haneul Jang[1*], Christophe Boesch[1,2], Roger Mundry[1], Simone D. Ban[2], Karline R.L. Janmaat[1,3]

[1] Department of Primatology, Max Planck Institute for Evolutionary Anthropology, Deutscher Platz 6, 04103 Leipzig, Germany

[2] Wild Chimpanzee Foundation, Abidjan, Côte d'Ivoire

[3] Institute for Biodiversity and Ecosystem Dynamics, Faculty of Science, University of Amsterdam, Amsterdam, The Netherlands

This file includes:

Supplementary Text:

Supplementary Methods

Full details of data collection.
Definition of tuber and mushroom patches.
Full details of statistical analyses.
Post-hoc analyses.

Supplementary Figures:

Figures S1 to S3

Supplementary Tables:

Tables S1 to S8

References for Supplementary Text

Supplementary Methods

Full details of data collection.

K.R.L.J and S.B.D conducted continuous focal follows[1] of adult chimpanzee females from the South community (mean: 29 individuals, range: 23 to 40) in Taï National Park, Côte d'Ivoire (5° 45 N, 7° 07 W; see ref[2] for details of the study site) from dawn to dusk (mean duration per day ± standard deviation: 11.38 ± 1.07 hours, range: 7.17 to 13.43 hours) for continuous periods ranging from 4 to 8 weeks for each subject from 2009 to 2011. A total of 274 full observation days were collected for chimpanzees, and the mean number of observations per focal was 26 days (range: 20 to 128 days) over 3 years. H.J., with the help of K.R.L.J, conducted focal follows of adult Mbendjele women from one forest camp named Mbaso (mean: 47 individuals, range: 20 to 79) near the village of Djoubé at the Motaba River in the department of Likouala in the north-western region of the Republic of Congo (2° 28 N, 17° 26 E). We followed their daily foraging trips from the moment the focal woman left the camp until her return (mean duration per day ± standard deviation: 5.20 ± 2.43 hours, range: 0.45 to 11.83 hours), except when they asked for privacy. We followed the same focal women twice for 4 consecutive weeks in each of 2015 and 2016. During the 265 days, the focal women stayed in the camp for a mean of two days per month (range: 0 to 7 days per month depending on the individual) due to illness of the individual or her children. We followed the Mbendjele's daily trips not only when foraging in the forest but also when gardening and visiting villages for social purposes (Fig. S2A) but used only foraging trips to the forest in our analyses, to allow comparison with the chimpanzees. A total of 236 observation days were obtained, with the mean per focal being 48 days (range: 41 to 53 days) over 2 years. We collected behavioural data in combination with a Garmin hand-held Global Positioning System (GPS; Garmin 60Csx for chimpanzees and Garmin 62 for humans) and a voice recorder. Each focal subject's daily routes were recorded with the GPS track log function and were cleaned using R (see Appendix in ref[3]). Age of independence was defined as the age at which the individuals begin travelling and foraging independently which happens at around nine years of age for chimpanzees and around five years for the Mbendjele, respectively. The tree density data for Djoubé forest were collected with the help

of research assistants. The research assistants walked through one big habitat plot sized one hectare and 18 small habitat plots sized 20 x 20 m which were randomly selected in the forest areas, and marked all trees with a DBH larger than 10 cm in the plots.

Inter-observer reliability for observations of human foragers was based on K.R.L.J and H.J. simultaneously following an individual, on eight sampling days. From observations made by H.J., we randomly selected 40 behaviours which fell into the four categories of collection of food, inspection of food, resting, or moving (ten behaviours per category), as well as ten additional behaviours which did not belong to any of these four categories. From the observations made by K.R.L.J, we then selected the 50 behaviours which had been recorded closest in time to each of the 50 behaviours selected from the observations by H.J., and compared 1) whether the behavioural category matched, and 2) observed group size was the same. This revealed a high correlation coefficient for group size ($r_s = 0.94$) and a large kappa for the occurrence of collection, inspection, resting, moving, and other behaviours within sampling days [1] ($k = 0.92$). Kappa was calculated in R (version 3.5.0) [4] using the package 'irr'[5] (version 0.84). Details of inter-observer reliability tests for chimpanzee data are provided in the Appendix of ref[3].

Definition of tuber and mushroom patches.

To include tuber and mushroom patches in the departure or arrival locations of human travel trajectories, we defined patches using behavioural data to reflect the Mbendjele's perspective of what constituted a food patch and how they viewed the dispersion of resources (see ref[6]). We located the places where mushroom collection or tuber digging occurred and identified the species. Most mushroom and tuber species had patch sizes smaller than the GPS accuracy and thus were easily distinguishable and detectable. However, one species of yam-like tuber (*Dioscoreophyllum cumminsii*, called *'méla'* by the Mbendjele) usually occurred in large patches of up to 500 m in diameter along their major axis. We used a heuristic approach to define patches for this tuber species. First, a collecting bout of *Dioscoreophyllum cumminsii* (hereafter *'méla'*) was defined as ending when the focal woman switched from *méla* to another food species, or when the focal woman told other members of the foraging group or the researcher that she would move to another

place to find more *méla*. We calculated the distances between *méla* spots within each collecting bout and defined the maximum distance observed (82 m) as the threshold distance for a *méla* patch. We then iteratively assigned digging locations ≤ 82 meters apart as belonging to the same patch. We found a total of 56 patches where the Mbendjele women collected *méla* during our observation period. We constructed a minimum convex polygon (MCP) around each *méla* patch to delineate its circumference using the R package 'adehabitatHR'[7] (version 0.4.14) and added a buffer using the R package 'polyclip'[8] (version 1.6-1) of 10 m around each *méla* patch to account for GPS inaccuracy. When the focal woman moved into or out of a *méla* patch, we defined the intersection between the border of the patch (MCP plus buffer) and the travel track as the arrival or departure location, respectively.

Full details of statistical analyses.

We inspected the distribution of all predictors for being approximately symmetrically distributed. After that we log-transformed familiarity, maximum age, group size, and straight line distance to achieve roughly symmetrical distributions. All quantitative predictors were then z-transformed to a mean of 0 and standard deviation of 1 before fitting the model[9]. All models accounted for the random effects of the oldest individuals in a foraging group, resource species at arrival location, arrival location identity, and observation day (see Table S7 for the results regarding the random effects). Since our selection of focal models was arbitrary, we included the identity of the oldest individual in a foraging group as one of the random effects, instead of focal individual identity. For the two linearity models, we used Generalized Linear Mixed Models (GLMM)[10] with a beta error structure (treating linearity as a proportion of the maximum possible linearity, i.e., one) and logit link function [11–13]. GLMMs were implemented using the function 'glmmTMB' of the package 'glmmTMB' (version 0.2.2.0)[14] in R version 3.5.0[4]. For the travel speed model, we used a linear mixed model (LMM)[10] with a Gaussian error structure and identity link function. This was implemented using the function 'lmer' of the package 'lme4' (version 1.1-19)[15] in R version 3.5.0[4]. In the LMM, we included all theoretically identifiable random slopes of fixed effects within random effects (Table S7) to keep the type I error probability at the desired level of 0.05[16,17]. To avoid

overly complex models and non-convergence issues in GLMMs, we used simplified random slope structures (Table S7), and did not estimate parameters for correlations among random slopes and intercepts [18]. See the footnote of Table S7 for the number of levels per random effect. For the LMM we checked whether the assumptions of normally distributed and homogeneous residuals were fulfilled by visually inspecting a qqplot[19] and the residuals plotted against fitted values. Both indicated no obvious deviations from these assumptions. Over-dispersion was no issue in the two GLMMs (maximum dispersion parameter: 1.027). Model stability was determined by evaluating model estimates obtained after dropping levels of the random effects one at a time. This showed that estimates did not vary strongly. We also checked for collinearity among predictors[20] by deriving Variance Inflation Factors (VIFs) using the function 'vif' of the package 'car'[21], applied to standard linear models including only the fixed effects (excluding interaction terms); no considerable collinearity was detected (maximum VIF = 2.28). As an overall test of the effect of species we first compared each full model with a respective null model[16] which excluded species and interactions with it but was otherwise identical. We used a likelihood ratio test (R function 'anova'[22]) for this comparison. Only when a full-null model comparison revealed significance (P<0.05), did we discuss the results of the model with respect to individual test predictors[16,23]. The significance of individual predictors was assessed using likelihood ratio tests comparing the full with respective reduced models[17] (R function 'drop1'). All p-values were two-tailed. We considered P≤0.05 as significant. We determined 95% confidence intervals using the function 'simulate.glmmTMB' of the 'glmmTMB' package[14] or 'bootMer' of the 'lme4' package[15] in R. In case of a significant interaction between the factor 'species' and a covariate (e.g., familiarity), we conducted *post hoc* tests to infer whether the effect of the covariate differed from zero in either of the two species. These we based on the results of the model which shows the effect of the covariate (and a test for it) for the effect of the covariate with species being at its reference level (i.e., chimpanzees) and, after changing the reference level of the factor 'species' also for humans. All data were analyzed in R (version 3.5.0)[4].

***Post hoc* analyses.**

To better understand why Taï chimpanzees' travelled less linear and slower in more familiar areas, we conducted a set of post-hoc analyses with the same data set for chimpanzees that we used for the linearity and travel speed model (N = 626 trajectories, 5 subjects). First, we tested whether chimpanzees had higher frequencies of social interactions (using sub-group fusion as a proxy for the opportunity or need for social interactions) when they travelled in more familiar areas compared to less familiar areas, which could disturb their goal-directed travels to food locations. Second, we tested whether chimpanzees rested more often in familiar areas than in less familiar areas, which could distort the goal-directedness of travels. Third, we tested whether chimpanzees inspected possible food sources in familiar areas more frequently than in less familiar areas, which also could reduce the linearity of travel. We used Generalized Linear Mixed Models (GLMM)[24] with a Poisson error structure for the above three models. The results show that there was no significant effect of familiarity on the frequency of either fusion or resting (Table S5). Contrary to our prediction, we found that chimpanzees inspected more often in less familiar areas than in familiar areas (Table S5), perhaps because they spent less time in the former areas and therefore needed to update their knowledge about the fruiting state of trees more often. In addition, we tested whether the current satiety levels during travel were higher in familiar areas than in less familiar areas, which might reduce their motivation to travel linearly and rapidly to food locations. Compared to familiar areas, there might be fewer known and available food trees in less-familiar areas, and thus chimpanzees might have a higher motivation to travel efficiently toward a food source due to elevated hunger levels. To this end we used an LMM with a Gaussian error structure. The cumulative energy balance (see ref[25] for details of calculations) was used as a proxy for hunger level, and we tested whether the energy balance during travel was lower in less-familiar areas. However, there was no significant effect of familiarity on cumulative energy balance (estimate = 1.430, SE = 1.891, χ^2= 1.592, df=1, P = 0.207), suggesting that variation in hunger level cannot explain increased goal-directedness in less familiar areas.

Supplementary Figures

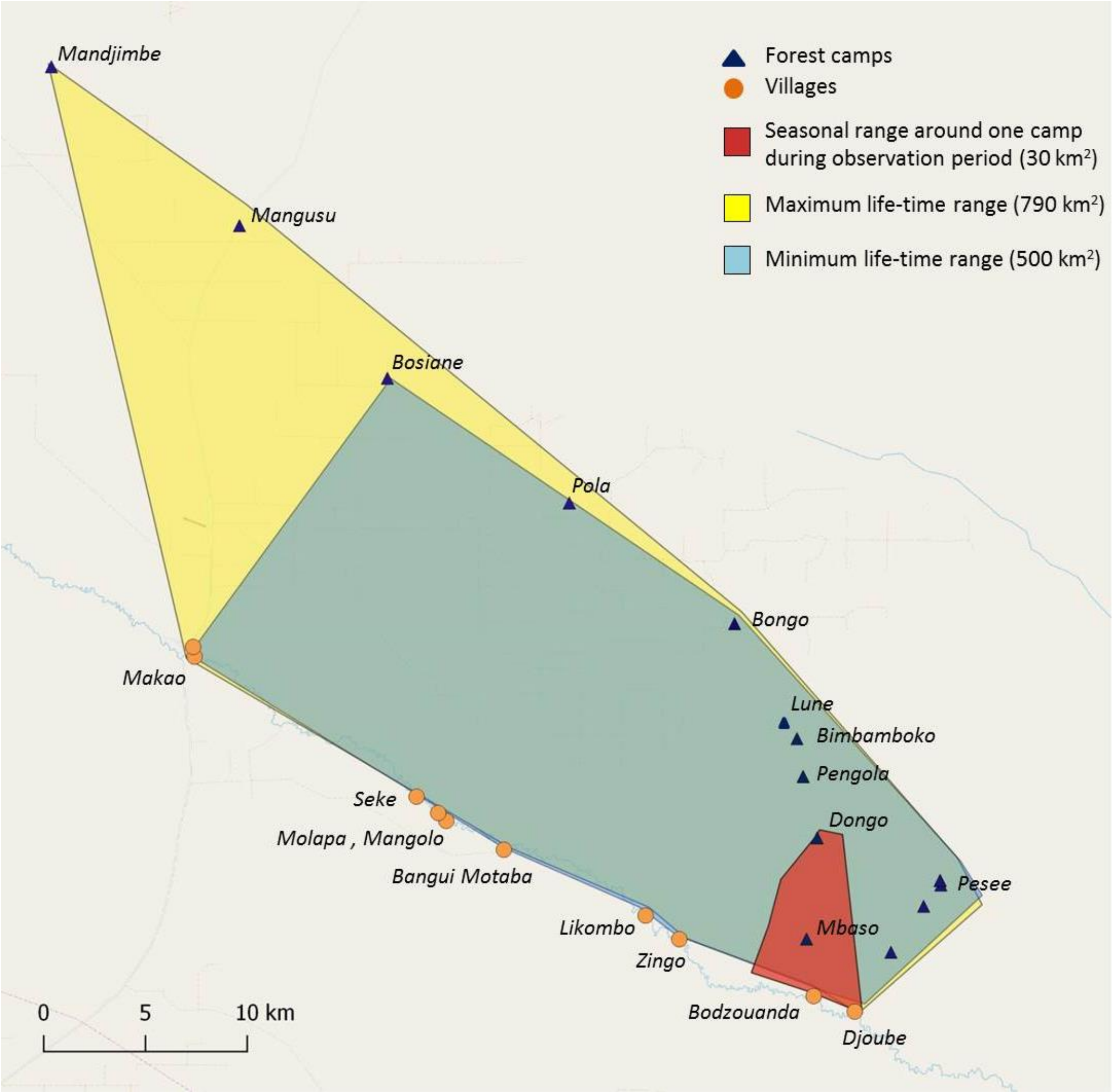

Figure S1. Illustration of the lifetime range and seasonal study range of one Mbendjele band. The *yellow polygon* represents the minimum convex polygon (MCP) for the maximum lifetime range of the oldest woman of the community; the *light blue polygon* represents the MCP for the minimum life-time range of a younger woman (aged approx. 30 years); the *red polygon* represents the MCP for the Mbendjele women's seasonal range around one forest camp for 236 days during the observation period, defined as the seasonal study range. From the interviews with seven adult Mbendjele women we obtained information on all the camps they visited during their life-time. We obtained information about the locations of those camps from logging company workers that had visited all the three northern camps, and K.R.L.J took the GPS coordinates of the other ones. Forest camps are depicted as *dark blue triangles* and villages as *orange circles*. The seasonal range (*red polygon*) was defined by using GPS track data of five Mbendjele women from the same band. This illustration was created with Quantum-GIS[25] (QGIS version 2.18.1).

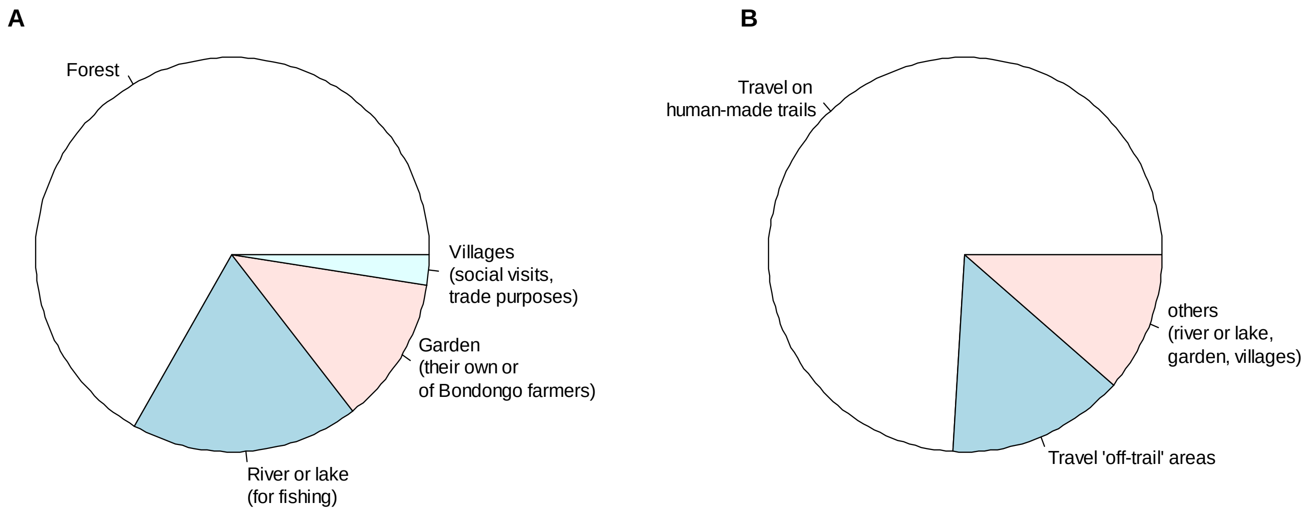

Figure S2. (A) Time spent foraging for food in the forest, river, gardens, and villages and (B) time spent travelling on human-made trails, off-trails, and in other areas. (A) The Mbendjele women spent 19 percent of their foraging time fishing, but fishing locations were not included in our analyses in order to make the data comparable with the chimpanzee data. Note that the five Mbendjele women (A) spent 70 % of their foraging time in the forest and (B) walked on human-made trails for 74 % of the total travel duration during focal follows (total of 1230 hours over 236 days).

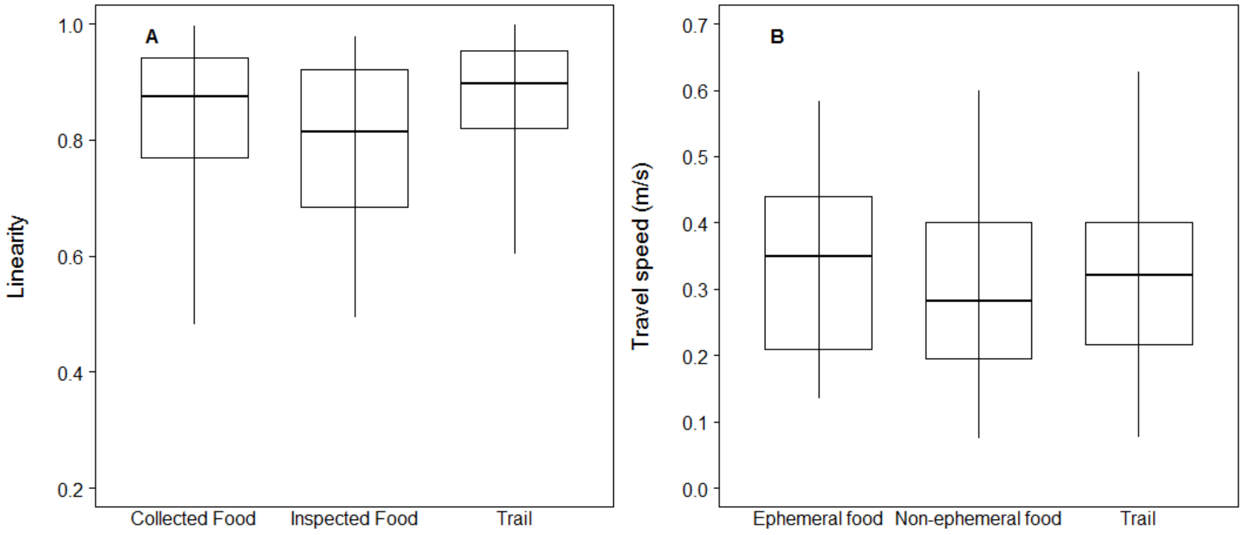

Figure S3. Distributions of (A) travel linearity and (B) travel speed, separately for the three different arrival location types. (A) The average travel linearity was slightly higher for 'return to a trail' trajectories compared to that of the other trajectories towards food-collection or food-inspection locations. However, the variation of the response was large within trajectories towards the same arrival location for all three of them, and this variation was considerably in excess of that between them. (B) The 'return to a trail' trajectories had a lower average travel speed than trajectories towards ephemeral foods.

Supplementary Tables

Table S1. Additional descriptive statistics of ranging behaviours of Mbendjele foragers and Taï chimpanzees

	**Mbendjele people (N_{women}=5, N_{days}=236)**	**Taï chimpanzees (N_{chimps}=5, N_{days}=274)**
**Mean** daily travel distance (km) using 1-minute intervals	4.52 ± 2.91 (range: 0.27 - 13.71)	4.36 ± 2.27 (range: 1.11 - 14.16)
**Mean** number of plant food locations per day	2.72 ± 2.11 (range: 1 - 13)	23.01 ± 13.50 (range: 4 - 83)
**Mean** distance (m) between successive plant food locations	422 ± 465 (range: 6 – 2772; $N_{distances}$=401)	153 ± 172 (range: 10 - 1898, $N_{distances}$=3764)
**Median** daily travel distance (km) using 30-minute intervals	2.57 (range: 0.2 - 11.81)	2.64 (range: 0.3 - 10.66)
**Mean** daily travel distance (km) using 30-minute intervals	3.24 ± 2.50	3.11 ± 1.80

Table S2. Linearity model 1 with group size: effects of seasonal familiarity with area, species, and group size upon travel linearity

Effect	Estimate	SE	CI_{lower}	CI_{upper}	χ^2	df	P
Full model with three-way interaction							
(Intercept)	1.329	0.064	1.210	1.458			*
Seasonal familiarity[1]	-0.177	0.037	-0.249	-0.105			*
Species (human)	0.241	0.122	0.011	0.488			*
Group size[2]	-0.146	0.078	-0.261	-0.030			*
**Straight line distance[3]**	**-0.175**	**0.045**	**-0.262**	**-0.086**	**11.581**	**1**	**<0.001**
**Female ratio[4]**	**-0.137**	**0.054**	**-0.233**	**-0.040**	**6.722**	**1**	**0.011**
Arrival location type (food-inspection location)[5]	0.098	0.077	-0.042	0.244	2.302	2	0.200
Arrival location type (trail)[5]	0.147	0.152	-0.152	0.447			*
Species (human): Seasonal familiarity	0.325	0.080	0.163	0.487			*
Seasonal familiarity: Group size	-0.013	0.033	-0.080	0.055			*
Species (human): Group size	0.311	0.099	0.131	0.487			*
Familiarity: Species (human): Group size	-0.030	0.072	-0.168	0.112	0.173	1	0.677
Intermediate model: reduced model after dropping insignificant three-way interaction							
(Intercept)	1.328	0.066	1.196	1.477			*
Seasonal familiarity[1]	-0.179	0.037	-0.257	-0.103			*
Species (human)	0.249	0.122	0.022	0.465			*
Group size[2]	-0.144	0.074	-0.256	-0.032			*
**Straight line distance[3]**	**-0.175**	**0.045**	**-0.263**	**-0.085**	**11.590**	**1**	**<0.001**
**Female ratio[4]**	**-0.137**	**0.053**	**-0.230**	**-0.043**	**6.742**	**1**	**0.010**
Arrival location type (food-inspection location)[5]	0.097	0.076	-0.050	0.239	2.210	2	0.202
Arrival location type (trail)[5]	0.140	0.153	-0.174	0.432			*
**Species (human): Seasonal familiarity**	**0.316**	**0.077**	**0.171**	**0.471**	**17.234**	**1**	**<0.001**
Seasonal familiarity: Group size	-0.019	0.029	-0.077	0.041	0.421	1	0.517
**Species (human): Group size**	**0.304**	**0.095**	**0.114**	**0.490**	**10.236**	**1**	**0.001**
Final model: reduced model after dropping insignificant two-way interaction between familiarity and group size							
(Intercept)	1.332	0.066	1.197	1.467			*
Seasonal familiarity [1]	0.248	0.123	0.012	0.495			*
Species (human)	-0.174	0.036	-0.249	-0.100			*
Group size[2]	-0.140	0.071	-0.250	-0.024			*
**Straight line distance[3]**	**-0.174**	**0.045**	**-0.266**	**-0.089**	**11.507**	**1**	**<0.001**
**Female ratio[4]**	**-0.134**	**0.053**	**-0.233**	**-0.030**	**6.577**	**1**	**0.011**
Arrival location type (food-inspection location)[5]	0.096	0.075	-0.048	0.237	2.160	2	0.201
Arrival location type (trail)[5]	0.134	0.153	-0.142	0.427			*
**Species (human): Seasonal familiarity**	**0.304**	**0.074**	**0.157**	**0.452**	**17.019**	**1**	**<0.001**
**Species (human): Group size**	**0.296**	**0.094**	**0.123**	**0.477**	**9.897**	**1**	**0.002**

Statistically significant results appear in bold. CI: confidence limit

* Not shown because of having a very limited interpretation

[1,2,3] Log- and then z-transformed; mean ± SD of the log-transformed values: [1]5.03 ± 1.06, [2]1.08 ± 0.79, [3]4.50 ± 0.80

[4] Z-transformed; mean ± SD of the original values: 0.78 ± 0.26

[5] Arrival location type was dummy coded with ‘food-collection location' being the reference category; the indicated test refers to the overall effect of arrival location type

Table S3. Linearity model 2 with the maximum age: effects of seasonal familiarity with area, species, and maximum age upon travel linearity

Effect	Estimate	SE	CI_{lower}	CI_{upper}	χ^2	df	P
Full model with three-way interaction							
(Intercept)	1.364	0.074	1.231	1.509			*
Seasonal familiarity[1]	-0.170	0.036	-0.241	-0.097			*
Species (human)	0.232	0.125	0.011	0.457			*
Maximum age[2]	-0.094	0.087	-0.259	0.064			*
**Straight line distance[3]**	**-0.171**	**0.046**	**-0.255**	**-0.088**	**10.807**	**1**	**<0.001**
Female ratio[4]	-0.060	0.037	-0.128	0.014	2.634	1	0.106
Type of arrival location (food-inspection location)[5]	0.056	0.071	-0.080	0.197	1.210	2	0.430
Type of arrival location (trail)[5]	0.125	0.140	-0.145	0.413			*
Species (human): Seasonal familiarity	0.285	0.073	0.136	0.439			*
Seasonal familiarity: Maximum age	0.064	0.061	-0.067	0.187			*
Species (human): Maximum age	0.076	0.094	-0.110	0.266			*
Seasonal familiarity: Species (human): Maximum age	-0.091	0.071	-0.238	0.069	1.634	1	0.200
Intermediate model: reduced model after dropping insignificant three-way interaction							
(Intercept)	1.359	0.073	1.224	1.505			*
Seasonal familiarity[1]	-0.166	0.036	-0.241	-0.090			*
Species (human)	0.242	0.125	0.005	0.492			*
Maximum age[2]	-0.092	0.085	-0.240	0.075			*
**Straight line distance[3]**	**-0.175**	**0.045**	**-0.266**	**-0.089**	**11.369**	**1**	**<0.001**
Female ratio[4]	-0.057	0.037	-0.122	0.018	2.385	1	0.124
Type of arrival location (food-inspection location)[5]	0.058	0.071	-0.081	0.185	1.185	2	0.413
Type of arrival location (trail)[5]	0.118	0.139	-0.173	0.404			
**Species (human): Seasonal familiarity**	**0.289**	**0.073**	**0.123**	**0.445**	**15.652**	**1**	**<0.001**
Seasonal familiarity: Maximum age	-0.003	0.032	-0.068	0.062	0.009	1	0.923
Species (human): Maximum age	0.074	0.092	-0.115	0.246	0.603	1	0.427
Final model: reduced model after dropping insignificant two-way interactions							
(Intercept)	1.365	0.075	1.225	1.514			*
Seasonal familiarity[1]	-0.166	0.036	-0.232	-0.097			*
Species (human)	0.237	0.125	-0.004	0.468			*
Maximum age[2]	-0.031	0.039	-0.105	0.042	0.559	1	0.438
**Straight line distance[3]**	**-0.173**	**0.045**	**-0.262**	**-0.087**	**11.121**	**1**	**<0.001**
Female ratio[4]	-0.056	0.037	-0.124	0.016	2.271	1	0.133
Type of arrival location (food-inspection location)[5]	0.059	0.071	-0.079	0.201	1.226	2	0.403
Type of arrival location (trail)[5]	0.119	0.139	-0.176	0.376			*
**Species (human): Seasonal familiarity**	**0.288**	**0.073**	**0.146**	**0.434**	**15.843**	**1**	**<0.001**

Statistically significant results appear in bold. CI: confidence limit

* Not shown because of to having a very limited interpretation

[1,2,3] Log- and then z-transformed; mean ± SD of the log-transformed values: [1]5.03 ± 1.06, [2]3.67 ± 0.20, [3]4.50 ± 0.80

[4] Z-transformed; mean ± SD of the original values: 0.78 ± 0.26

[5] Arrival location type was dummy coded with ‘food-collection location' being the reference category; the indicated test refers to the overall effect of arrival location type

Table S4. Travel speed model: effects of seasonal familiarity with area, species, and maximum age upon travel speed

Effect	Estimate	SE	CI_{lower}	CI_{upper}	χ^2	df	P
Full model with three-way interaction							
(Intercept)	0.398	0.028	0.343	0.458			*
Seasonal familiarity[1]	-0.053	0.009	-0.072	-0.035			*
Species (human)	-0.066	0.033	-0.133	-0.001			*
Maximum age[2]	0.024	0.023	-0.024	0.072			*
**Straight line distance[3]**	**0.038**	**0.010**	**0.017**	**0.059**	**8.537**	**1**	**0.003**
Female ratio[4]	-0.007	0.009	-0.025	0.012	0.633	1	0.426
Type of arrival location (non-ephemeral food)[5]	0.005	0.021	-0.038	0.047	0.422	2	0.810
Type of arrival location (trail)[5]	0.020	0.031	-0.041	0.084			*
Group size[6]	-0.005	0.009	-0.023	0.014	0.292	1	0.589
Species (human): Seasonal familiarity	0.041	0.018	0.005	0.076			*
Seasonal familiarity: Maximum age	0.006	0.013	-0.019	0.034			*
Species (human): Maximum age	-0.014	0.025	-0.067	0.036			*
Seasonal familiarity: Species (human): Maximum age	-0.022	0.015	-0.053	0.007	1.887	1	0.170
Intermediate model: reduced model after dropping insignificant three-way interaction							
(Intercept)	0.397	0.028	0.342	0.454			*
Seasonal familiarity[1]	-0.054	0.010	-0.074	-0.034			*
Species (human)	-0.064	0.033	-0.133	0.002			*
Maximum age[2]	0.021	0.022	-0.023	0.066			*
**Straight line distance[3]**	**0.037**	**0.010**	**0.019**	**0.058**	**8.778**	**1**	**0.003**
Female ratio[4]	-0.006	0.009	-0.023	0.012	0.532	1	0.466
Type of arrival location (non-ephemeral food)[5]	0.004	0.021	-0.037	0.046	0.375	2	0.829
Type of arrival location (trail)[5]	0.018	0.031	-0.044	0.079			*
Group size[6]	-0.005	0.009	-0.022	0.012	0.314	1	0.575
**Species (human): Seasonal familiarity**	**0.043**	**0.019**	**0.007**	**0.080**	**4.259**	**1**	**0.039**
Seasonal familiarity: Maximum age	-0.010	0.007	-0.023	0.004	1.992	1	0.158
Species (human): Maximum age	-0.011	0.024	-0.061	0.038	0.219	1	0.640
Final model: reduced model after dropping insignificant two-way interactions							
(Intercept)	0.394	0.028	0.338	0.450			*
Seasonal familiarity[1]	-0.053	0.010	-0.072	-0.033			*
Species (human)	-0.062	0.032	-0.129	0.004			*
Maximum age[2]	0.011	0.010	-0.009	0.032	1.096	1	0.295
**Straight line distance[3]**	**0.037**	**0.009**	**0.019**	**0.055**	**9.066**	**1**	**0.003**
Female ratio[4]	-0.006	0.009	-0.024	0.011	0.449	1	0.503
Type of arrival location (non-ephemeral food)[5]	0.005	0.022	-0.039	0.047	0.329	2	0.848
Type of arrival location (trail)[5]	0.018	0.031	-0.047	0.079			*
Group size[6]	-0.004	0.009	-0.021	0.013	0.178	1	0.673
**Species (human): Seasonal familiarity**	**0.045**	**0.019**	**0.006**	**0.082**	**4.431**	**1**	**0.035**

Statistically significant results appear in bold. CI: confidence limit

* Not shown because of having a very limited interpretation

[1,2,3,6] Log- and then z-transformed; mean ± SD of log-transformed values: [1]5.03 ± 1.06, [2]3.67 ± 0.20, [3]4.50 ± 0.80, [6]1.08 ± 0.79

[4] Z-transformed; mean ± SD of the original values: 0.78 ± 0.26

[5] Arrival location type was dummy coded with 'ephemeral food' being the reference category; the indicated test refers to the overall effect of arrival location type

Table S5. Effects of familiarity with an area on the frequencies of group fusion, resting, and food-monitoring of chimpanzees

Response	Effect	Estimate	SE	df	P
Fusion frequency	(Intercept)	-8.172	0.140		*
	Familiarity[1]	-0.179	0.117	1	0.144
	**Initial group size[2]**	**-0.245**	**0.102**	**1**	**0.022**
Resting frequency	(Intercept)	-0.885	0.219		*
	Familiarity[1]	0.119	0.091	1	0.187
Monitoring frequency	(Intercept)	-6.217	0.228		*
	**Familiarity[1]**	**-0.249**	**0.110**	**1**	**0.023**

Statistically significant results appear in bold

* Not shown because of having a very limited interpretation

[1,2] Log- and then z-transformed; mean ± SD of the log-transformed values: [1]5.03 ± 1.06, [2]1.09 ± 0.79

Table S6. Departure and arrival location types of travel trajectories in Taï chimpanzees and Mbendjele foragers

	Departure location	Arrival location
**Taï chimpanzees**	fruit collection tree	fruit collection tree
	fruit inspection tree	fruit inspection tree
	chimpanzees ate leaves of the following species: *Haloplegia azurea, Cola heterophylla, Entandrophragma cylindricum, Hypselodelphys violacead, Elaeis guineensis, Afzelia bella, Sterculia oblonga, Trichoscypha arborea*	
**Mbendjele people**	fruit collection tree	fruit collection tree
	fruit inspection tree	fruit inspection tree
	tuber collection patch	tuber collection patch
	mushroom collection patch	mushroom collection patch
	trail location where she left a trail	trail location where she reached a trail

Table S7. *Post hoc* travel speed model with an additional two-way interaction between species and group size

Effect	Estimate	SE	CI_{lower}	CI_{upper}	χ^2	df	P
Full model with a three-way interaction and a two-way interaction between species and group size							
(Intercept)	0.398	0.027	0.343	0.452			*
Seasonal familiarity[1]	-0.053	0.009	-0.070	-0.033			*
Species (human)	-0.072	0.032	-0.131	-0.005			*
Maximum age[2]	0.031	0.022	-0.014	0.076			*
**Straight line distance[3]**	**0.037**	**0.010**	**0.017**	**0.057**	**8.305**	**1**	**0.004**
Female ratio[4]	-0.014	0.010	-0.033	0.005	1.647	1	0.199
Type of arrival location (non-ephemeral food)[5]	0.006	0.023	-0.035	0.050	0.544	2	0.762
Type of arrival location (trail)[5]	0.023	0.031	-0.038	0.078			*
Group size[6]	-0.019	0.012	-0.042	0.003			*
**Species (human): Group size**	**0.032**	**0.018**	**-0.003**	**0.066**	**2.989**	**1**	**0.084**
Species (human): Seasonal familiarity	0.041	0.017	0.006	0.075			*
Seasonal familiarity: Maximum age	0.005	0.013	-0.021	0.033			*
Species (human): Maximum age	-0.025	0.024	-0.078	0.021			*
Seasonal familiarity: Species (human): Maximum age	-0.022	0.015	-0.052	0.008	1.700	1	0.192
Reduced model after dropping insignificant three-way interaction and two-way interaction							
(Intercept)	0.391	0.027	0.336	0.445			*
Seasonal familiarity[1]	-0.054	0.010	-0.073	-0.033			*
Species (human)	-0.065	0.031	-0.128	0.006			*
Maximum age[2]	0.009	0.009	-0.010	0.028	0.804	1	0.370
**Straight line distance[3]**	**0.036**	**0.009**	0.017	0.054	**8.781**	**1**	**0.003**
Female ratio[4]	-0.012	0.010	-0.033	0.007	1.373	1	0.241
Type of arrival location (non-ephemeral food)[5]	0.006	0.022	-0.035	0.047	0.420	2	0.811
Type of arrival location (trail)[5]	0.021	0.032	-0.043	0.079			*
Group size[6]	-0.015	0.011	-0.039	0.007			*
Seasonal familiarity: Group size	0.027	0.017	-0.008	0.063	2.436	1	0.119
**Species (human): Seasonal familiarity**	**0.045**	**0.029**	0.010	0.084	**4.562**	**1**	**0.033**

Statistically significant results appear in bold. CI: confidence limit

* Not shown because of having a very limited interpretation

[1,2,3,6] Log- and then z-transformed; mean ± SD of log-transformed values: [1]5.03 ± 1.06, [2]3.67 ± 0.20, [3]4.50 ± 0.80, [6]1.08 ± 0.79

[4] Z-transformed; mean ± SD of the original values: 0.78 ± 0.26

[5] Arrival location type was dummy coded with 'ephemeral food' being the reference category; the indicated test refers to the overall effect of arrival location type

Table S8. Random slopes in each full model and estimated variance components (standard deviations) for the random effects and residuals from the full models

Random effects	Term[2]	Standard Deviation
1) Linearity model 1 with group size (Table 2, S2)[1]		
Arrival location ID	(Intercept)	0.000
Observation day ID	(Intercept)	0.143
Resource species at arrival location	Straight line distance	0.165
Resource species at arrival location	(Intercept)	0.091
Oldest individual ID	(Intercept)	0.071
2) Linearity model 2 with the maximum age (Table S3)[1]		
Arrival location ID	(Intercept)	0.000
Observation day ID	(Intercept)	0.141
Resource species at arrival location	Straight line distance	0.163
Resource species at arrival location	(Intercept)	0.073
Oldest individual ID	(Intercept)	0.116
3) Travel speed model with the maximum age (Table 2, S4)[1]		
Arrival location ID	(Intercept)	0.080
Observation day ID	(Intercept)	0.042
Resource species at arrival location	Straight line distance	0.000
Resource species at arrival location	Familiarity	0.016
Resource species at arrival location	(Intercept)	0.006
Oldest individual ID	Familiarity: Maximum age	0.000
Oldest individual ID	Straight line distance	0.027
Oldest individual ID	Group size	0.000
Oldest individual ID	Maximum age	0.000
Oldest individual ID	Female ratio	0.000
Oldest individual ID	Familiarity	0.008
Oldest individual ID	(Intercept)	0.048
Residual		0.135

[1] Number of observations = 877; number of levels of random effects: Arrival location ID = 720; Observation day ID = 301; Resource species at arrival location= 89; ID of oldest individual = 27

[2] The column 'term' specifies whether the row refers to a random intercepts or random slopes component.

Supplementary References

1. Martin, P. & Bateson, P. P. G. *Measuring Behaviour: An Introductory Guide*. (Cambridge Univ. Press, 2009).
2. Boesch, C. & Boesch-Achermann, H. *The Chimpanzees of the Taï Forest: Behavioural Ecology and Evolution*. (Oxford Univ. Press, 2000).
3. Janmaat, K. R. L., Ban, S. D. & Boesch, C. Chimpanzees use long-term spatial memory to monitor large fruit trees and remember feeding experiences across seasons. *Anim. Behav.* **86**, 1183–1205 (2013).
4. R Core Team. R: A Language and Environment for Statistical Computing. R Foundation for Statistical Computing, Vienna, Austria URL. (2018).
5. Gamer, M., Lemon, J., Gamer, M. M., Robinson, A. & Kendall's, W. Package 'irr'. *Var. Coeff. Interrater Reliab. Agreem.* (2012).
6. Vogel, E. R. & Janson, C. H. Quantifying Primate Food Distribution and Abundance for Socioecological Studies: An Objective Consumer-centered Method. *Int. J. Primatol.* **32**, 737–754 (2011).
7. Calenge, C. Home range estimation in R: the adehabitatHR package. *Off. Natl. Cl. Faune Sauvage St. Benoist Auffargis Fr.* (2011).
8. Johnson, A., Baddeley, A., Ripley, B. D. & Hornik, K. Package 'polyclip'. (2017).
9. Schielzeth, H. Simple means to improve the interpretability of regression coefficients. *Methods Ecol. Evol.* **1**, 103–113 (2010).
10. Baayen, R. H. *Analyzing Linguistic Data: A Practical Introduction to Statistics using R*. (Cambridge Univ. Press, 2008).
11. McCullagh, P. & Nelder, J. A. *Generalized Linear Models, Second Edition*. (CRC Press, 1989).
12. Bolker, B. M. *Ecological Models and Data in R*. (Princeton University Press, 2008).
13. Ferrari, S. & Cribari-Neto, F. Beta Regression for Modelling Rates and Proportions. *J. Appl. Stat.* **31**, 799–815 (2004).
14. Brooks, M. E. *et al.* glmmTMB balances speed and flexibility among packages for zero-inflated generalized linear mixed modeling. *R J.* **9**, 378–400 (2017).
15. Bates, D., Mächler, M., Bolker, B. & Walker, S. Fitting Linear Mixed-Effects Models using lme4. *J. Stat. Softw.* **67**, 1–48 (2015).
16. Forstmeier, W. & Schielzeth, H. Cryptic multiple hypotheses testing in linear models: overestimated effect sizes and the winner's curse. *Behav. Ecol. Sociobiol.* **65**, 47–55 (2011).

17. Barr, D. J., Levy, R., Scheepers, C. & Tily, H. J. Random effects structure for confirmatory hypothesis testing: Keep it maximal. *J. Mem. Lang.* **68**, 255–278 (2013).

18. Matuschek, H., Kliegl, R., Vasishth, S., Baayen, H. & Bates, D. Balancing Type I error and power in linear mixed models. *J. Mem. Lang.* **94**, 305–315 (2017).

19. Field, A. *Discovering Statistics Using SPSS*. (Sage Publications. London, 2009).

20. Quinn, G. P. & Keough, M. J. *Experimental Design and Data Analysis for Biologists*. (Cambridge Univ. Press, 2002).

21. Fox, J. *et al.* Package 'car'. (2017).

22. Dobson, A. J. & Barnett, A. *An Introduction to Generalized Linear Models.* (Chapman & Hall/CRC, 2008).

23. Mundry, R. Statistical issues and assumptions of phylogenetic generalized least squares. in *Modern Phylogenetic Comparative Methods and Their Application in Evolutionary Biology* 131–153 (Springer, Berlin, Heidelberg, 2014).

24. Bolker, B. M. *et al.* Generalized linear mixed models: a practical guide for ecology and evolution. *Trends Ecol. Evol.* **24**, 127–135 (2009).

25. Janmaat, K. R. L., Polansky, L., Ban, S. D. & Boesch, C. Wild chimpanzees plan their breakfast time, type, and location. *Proc. Natl. Acad. Sci.* **111**, 16343–16348 (2014).

25. QGIS Development Team (2018). QGIS Geographic Information System. Open Source Geospatial Foundation Project. http://qgis.osgeo.org
